# Antitumor effects of radionuclide treatment using α-emitting *meta*-^211^At-astato-benzylguanidine in a PC12 pheochromocytoma model

**DOI:** 10.1007/s00259-017-3919-6

**Published:** 2018-01-19

**Authors:** Yasuhiro Ohshima, Hitomi Sudo, Shigeki Watanabe, Kotaro Nagatsu, Atsushi B. Tsuji, Tetsuya Sakashita, Yoichi M. Ito, Keiichiro Yoshinaga, Tatsuya Higashi, Noriko S. Ishioka

**Affiliations:** 1 Department of Radiation-Applied Biology Research, Quantum Beam Science Research Directorate, National Institutes for Quantum and Radiological Science and Technology, 1233 Watanukimachi, Takasaki-shi, Gunma 370-1292 Japan; 2 Department of Molecular Imaging and Theranostics, National Institutes for Quantum and Radiological Science and Technology, National Institute of Radiological Sciences, 4-9-1 Anagawa, Inage-ku, Chiba, 263-8555 Japan; 3 Department of Radiopharmaceuticals Development, National Institutes for Quantum and Radiological Science and Technology, National Institute of Radiological Sciences, 4-9-1 Anagawa, Inage-ku, Chiba, 263-8555 Japan; 4 Department of Biostatistics, Hokkaido University Graduate School of Medicine, Kita 15, Nishi 7, Kita-ku, Sapporo, 060-8638 Japan; 5 Diagnostic and Therapeutic Nuclear Medicine, National Institutes for Quantum and Radiological Science and Technology, National Institute of Radiological Sciences, 4-9-1 Anagawa, Inage-ku, Chiba, 263-8555 Japan

**Keywords:** α-Emitter, *meta*-^211^At-astato-benzylguanidine, Norepinephrine transporter, Pheochromocytoma, Radionuclide therapy

## Abstract

**Purpose:**

Therapeutic options for patients with malignant pheochromocytoma are currently limited, and therefore new treatment approaches are being sought. Targeted radionuclide therapy provides tumor-specific systemic treatments. The β-emitting radiopharmaceutical *meta*-^131^I-iodo-benzylguanidine (^131^I-MIBG) provides limited survival benefits and has adverse effects. A new generation of radionuclides for therapy using α-particles including *meta*-^211^At-astato-benzylguanidine (^211^At-MABG) are expected to have strong therapeutic effects with minimal side effects. However, this possibility has not been evaluated in an animal model of pheochromocytoma. We aimed to evaluate the therapeutic effects of the α-emitter ^211^At-MABG in a pheochromocytoma model.

**Methods:**

We evaluated tumor volume-reducing effects of ^211^At-MABG using rat pheochromocytoma cell line PC12 tumor-bearing mice. PC12 tumor-bearing mice received intravenous injections of ^211^At-MABG (0.28, 0.56, 1.11, 1.85, 3.70 and 5.55 MBq; five mice per group). Tumor volumes were evaluated for 8 weeks after ^211^At-MABG administration. The control group of ten mice received phosphate-buffered saline.

**Results:**

The ^211^At-MABG-treated mice showed significantly lower relative tumor growth during the first 38 days than the control mice. The relative tumor volumes on day 21 were 509.2% ± 169.1% in the control mice and 9.6% ± 5.5% in the mice receiving 0.56 MBq (*p* < 0.01). In addition, the mice treated with 0.28, 0.56 and 1.11 MBq of ^211^At-MABG showed only a temporary weight reduction, with recovery in weight by day 10.

**Conclusion:**

^211^At-MABG exhibited a strong tumor volume-reducing effect in a mouse model of pheochromocytoma without weight reduction. Therefore, ^211^At-MABG might be an effective therapeutic agent for the treatment of malignant pheochromocytoma.

**Electronic supplementary material:**

The online version of this article (10.1007/s00259-017-3919-6) contains supplementary material, which is available to authorized users.

## Introduction

Approximately 10% to 25% of patients with pheochromocytoma have systemic metastasis known as malignant pheochromocytoma [1–3]. Tumor mass effects and catecholamine induce several pathological conditions [1, 4, 5]. These are associated with mortality. Patients with malignant pheochromocytoma have limited treatment options that include chemotherapy with cyclophosphamide, vincristine, and dacarbazine (CVD) [6, 7], and radionuclide therapy using β-emitting *meta*-^131^I-iodo-benzylguanidine (^131^I-MIBG). The effects of CVD are of limited duration [6, 8]. Of these treatments, ^131^I-MIBG has been shown to prolong survival [8].

Targeted radionuclide therapy (TRT) is a target-specific systemic therapy with a simple cytotoxic mechanism that directly targets cells such as those with DNA damage from radiation [9]. ^131^I-MIBG is a false analog of norepinephrine and is therefore taken into the pheochromocytoma cell via the uptake-1 mechanism [10, 11]. ^131^I-MIBG, because of the cytotoxic effects of β-radiation, can improve survival in patients with malignant pheochromocytoma [12–14]. However, even with high doses of ^131^I-MIBG, survival is still limited and ^131^I-MIBG is associated with radiation-induced side effects such as bone marrow suppression and lung injury [15]. Therefore, new therapeutic approaches are required to treat malignant pheochromocytoma.

A new generation of TRT involves the use of α-particles. The α-particle is exclusively cytotoxic and not affected by many of the limitations associated with conventional chemotherapy and radionuclide therapy. The α-particle has high mean energy deposition (linear energy transfer, LET) and a limited range in tissue, resulting in strong therapeutic effects with minimal side effects [16]. Theoretically, ^211^At-MABG should be more effective and have fewer side effects.

The therapeutic applications of α-emitters have mainly focused on ^211^At, ^233^Ra, ^213^Bi and ^225^Ac [16, 17]. For our purposes, we required an α-emitter-labeled ligand of the norepinephrine transporter. To maintain the affinity of a benzylguanidine analog for the norepinephrine transporter, we had to use an α-emitter which has similar characteristics to ^131^I. Concerning the therapeutic applications of α-emitters, ^211^At is a halogen and has similar characteristics to ^131^I [18]. Therefore, ^211^At is suitable for labeling of a benzylguanidine analog with an α-emitter, and ^211^At-MABG will have characteristics similar to those of ^131^I-MIBG [19]. A previous study showed in vitro cytotoxicity in neuroblastoma cells [20]. However, to date there have been no studies looking at the therapeutic effect of ^211^At-MABG in neuroblastoma and pheochromocytoma in vivo in animal models*.* The purpose of the present study was to investigate the therapeutic effects of ^211^At-MABG in a pheochromocytoma model both in vitro and in vivo.

## Materials and methods

### Production of ^211^At and radiosynthesis of ^211^At-MABG

^211^At was produced and recovered as described previously [19, 21] (Supplementary material). The emitted radioactivity of ^211^At was 22.1–93.2 MBq at the end of bombardment, and the radiochemical purity of ^211^At was more than 99.9% at the end of recovery. Benzylguanidine analog was labeled with ^211^At according to a previously published method [19] with slight modification (see a detailed description in the Supplementary material). A high-purity germanium detector was used to measure radioactivity. Radiochemical purity of ^211^At-MABG was estimated using reverse-phase radio-high-performance liquid chromatography (radio-HPLC).

### Cell culture

PC12 rat pheochromocytoma cells (Japanese Collection of Research Bioresources Cell Bank, Osaka, Japan) that have high norepinephrine transporter expression [22] were cultured in RPMI-1640 (Wako Pure Chemical Industries, Osaka, Japan) containing 10% horse serum (Thermo Fisher Scientific, Waltham, MA) and 5% fetal bovine serum (Serum Source International, Charlotte, NC). The cells were cultured at 37 °C in humidified air containing 5% CO_2_.

### Cell survival assay

PC12 cells (1 × 10^6^ cells) were incubated with 0, 0.2, 0.6, 2.0, 6.0 and 20.0 kBq/mL of ^211^At-MABG for 24 h. The cells were then washed with phosphate-buffered saline (PBS), suspended in growth medium, and seeded at 400 cells/well in a 96-well plate. After incubation for 14 days, the cells were incubated with 0.5 mg/mL of 3-(4,5-dimethylthiazol-2-yl)-2,5-diphenyltetrazolium bromide (MTT) for 4 h at 37 °C. Absorbance at 590 nm was measured using a plate reader (VMax; Molecular Devices, Sunnyvale, CA). Rates of cell survival were normalized to the absorbance of control cultures treated with 0 kBq/mL.

### DNA double-strand break assay

PC12 cells (1 × 10^6^ cells) were incubated with 0, 0.6, 2.0 and 6.0 kBq/mL of ^211^At-MABG for 24 h. The neutral comet assay was used to detect DNA double-strand breaks (DSB) using a CometAssay kit (Trevigen, Gaithersburg, MD) according to the manufacturer’s instructions. Comet tails were stained with SYBR Green and analyzed using a fluorescent microscope.

### Pheochromocytoma mouse model

The animal experimental protocol was approved by the Animal Care and Use Committees of our institutions, and all animal experiments were conducted in accordance with the institutional guidelines regarding animal care and handling. PC12 cells (3 × 10^6^) were subcutaneously inoculated into the right hind limb of female BALB/c-nu/nu mice at 5 weeks of age (CLEA Japan, Tokyo, Japan) under isoflurane anesthesia.

### Biodistribution study

PC12 tumor-bearing mice (five mice per time point) were injected with 100 kBq of ^211^At-MABG in 100 μL of PBS into a tail vein. The mice were killed at 1, 3, 6, 12 and 24 h after ^211^At-MABG administration. Blood, tumor, and organs of interest were dissected and weighed, and radioactivity was measured using a γ-counter (ARC-7001; Aloka, Tokyo, Japan). The radioactivity of organs and tissues except the thyroid is presented as the percentage injected radioactivity dose per gram (% ID/g), and that of the thyroid as percentage injected radioactivity dose (% ID) [23].

### Dosimetry

The mean doses per unit of injected activity (grays per megabecquerel) of α-disintegrations from ^211^At absorbed by each organ and tumor were estimated according to the standard method using the Medical Internal Radiation Dose formula [24, 25] (Supplementary material).

### Monitoring tumor volume and body weight after ^211^At-MABG treatment

When the PC12 tumor volumes had reached approximately 50 mm^3^, the mice (body weight 20.89 ± 1.30 g) were injected intravenously with ^211^At-MABG (0.28, 0.56, 1.11, 1.85, 3.70 and 5.55 MBq; five mice per dose) or PBS (ten mice). Tumor size and body weight were measured at least twice a week for 8 weeks after ^211^At-MABG administration. Tumor size was measured using a digital caliper, and tumor volume was calculated using the formula: tumor volume (mm^3^) = (length × width^2^)/2.

Body weight loss is considered to be one of the major radiation-related side effects of radiopharmaceuticals. Therefore, we evaluated the change in body weight of the mice as a marker of radiation-related side effects [26].

### Primary endpoint after ^211^At-MABG treatment

When body weight loss was more than 20% compared with that at baseline (day 0), signs of a moribund state were observed, or the tumor volume had reached more than 800 mm^3^, the mouse was killed humanely by isoflurane inhalation, and the tumor was resected for histological analysis. For Kaplan-Meier survival analysis, a tumor volume of 500 mm^3^ was considered the endpoint in addition to a body weight loss of more than 20%.

### Pathological analysis: hematoxylin and eosin staining and immunohistochemical staining

For analysis of temporal histological change in tumors, subcutaneous PC12 tumors were resected from the mice on days 1, 3 and 7 after administration of 1.11 MBq of ^211^At-MABG (three mice per time point). For analysis of dose-dependent tumor volume reduction, PC12 tumors from mice killed on day 3 or 4 after injection of 1.85, 3.70 and 5.55 MBq of ^211^At-MABG were evaluated as described above.

The resected tumors were fixed in 10% neutral-buffered formalin and embedded in paraffin. The tumor sections (1 μm thick) were deparaffinized and stained with hematoxylin and eosin (H&E). Immunohistochemical staining for Ki-67 was performed using rabbit anti-Ki-67 (Abcam, Cambridge, MA) and an anti-rabbit HRP/DAB detection kit (Abcam) according to the manufacturer’s instructions [27].

To evaluate systemic toxicity of ^211^At-MABG, histological changes in the bone marrow, adrenal glands, and heart of the mice were analyzed on H&E-stained sections. These organs were resected from the mice at the same time as the tumors. The organs were fixed and embedded in paraffin as described above. These sections (1 μm thick) were deparaffinized and stained with H&E. Images were obtained using a NanoZoomer S60 virtual slide scanner (Hamamatsu Photonics, Shizuoka, Japan).

### Statistical analysis

Continuous measures are presented as means ± standard deviation. Data were analyzed by analysis of variance with Dunnett’s multiple comparison test. The survival curves for each treatment group were compared with that for the control group using the log-rank test [28]. A *p* value <0.05 was considered statistically significant. Statistical calculations were carried out using GraphPad Prism and Statcel 3.

## Results

### Chemical and biological characterization of ^211^At-MABG

The radio-HPLC analysis, cell uptake assay and inhibition assay were performed to confirm that the product was ^211^At-MABG. The retention time (*t*_R_) of the product was 19.1 min, which was close to that of nonradioactive MIBG (*t*_R_ = 18.7 min); Supplementary Fig. 1). The radiochemical yield after HPLC purification was 61.5 ± 14.4% (decay-corrected, *n* = 4) and the radiochemical purity was over 99.7%. The cell uptake assay showed that the product was rapidly transported into PC12 cells that have high norepinephrine transporter expression (Supplementary Fig. 2a). The inhibition assay showed that desipramine (DMI), a selective inhibitor of the norepinephrine transporter, and *dl*-norepinephrine significantly inhibited cell uptake of the product (*p* < 0.01, Supplementary Fig. 2b), and uptake was also significantly suppressed by incubation at 4 °C.

### In vitro tumor cell-damaging effects of ^211^At-MABG

**Fig. 1:**
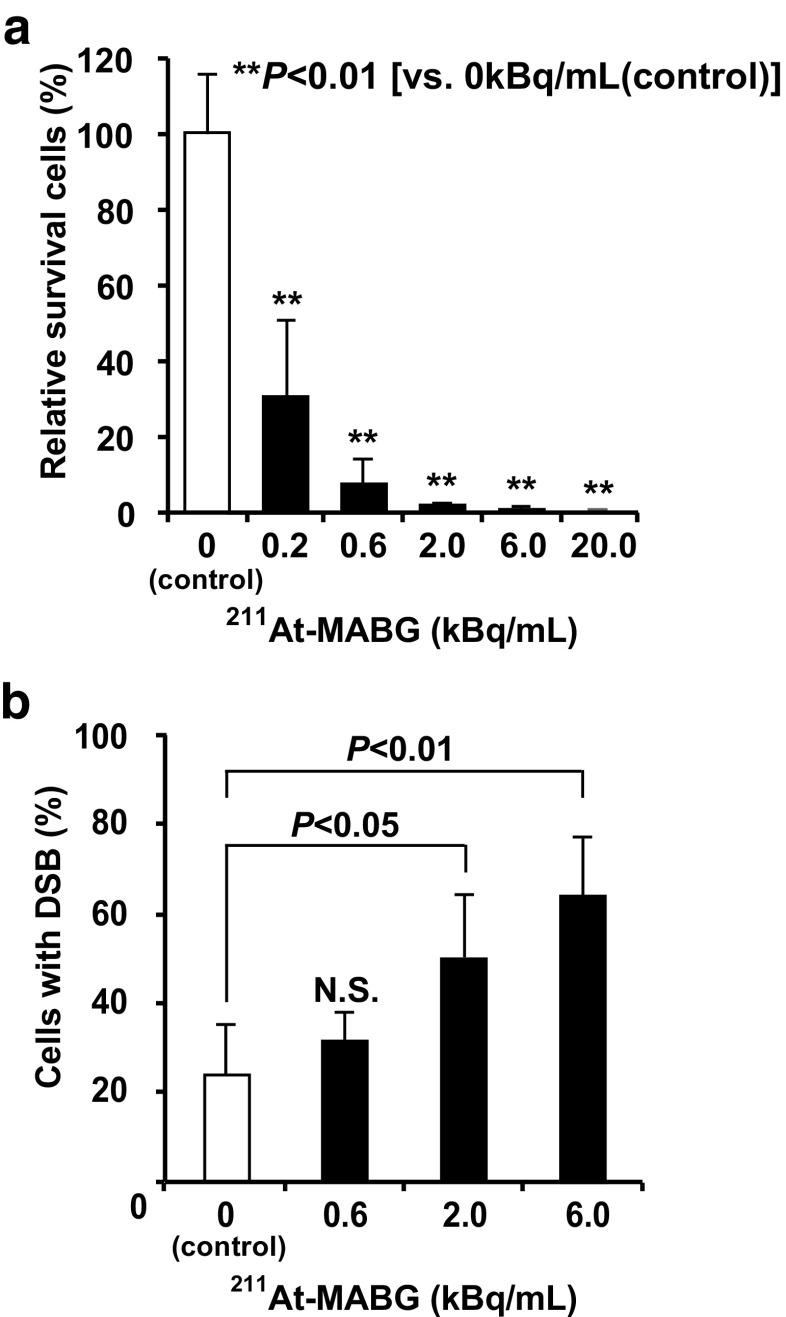
In vitro cytotoxicity of ^211^At-MABG. **a** Cell survival of PC12 pheochromocytoma cells after treatment with 0, 0.2, 0.6, 2.0, 6.0 and 20.0 kBq/mL of ^211^At-MABG. Cell survival was determined using the MTT assay and normalized to survival of cells without ^211^At-MABG treatment (0 kBq/mL, control). **p < 0.01 (vs. 0 kBq/mL, control). **b** Cells with DNA double-strand breaks (DSB) after treatment with 0, 0.6, 2.0 and 6.0 kBq/mL of ^211^At-MABG. **p < 0.01, *p < 0.05 (vs. 0 kBq/mL)

^211^At-MABG treatment dose-dependently suppressed survival of PC12 cells relative to control cells without ^211^At-MABG treatment, as shown using the MTT assay (*p* < 0.01,vs. control; Fig. 1a). ^211^At-MABG treatment induced lactate dehydrogenase (LDH) release (a cell death marker) from PC12 cells (*p* < 0.01,vs. control; Supplementary Fig. 3). ^211^At-MABG treatment dose-dependently increased the proportion of cells with DNA DSB and the percentages of cells with DNA DSB treated with 2.0 and 6.0 kBq/mL ^211^At-MABG were significantly higher than that in the control group (*p* < 0.05 for 2.0 kBq/mL, *p* < 0.01 for 6.0 kBq/mL; Fig. 1b).

### In vivo study

#### Biodistribution and dosimetry studies

Table 1 shows the biodistribution of ^211^At-MABG in PC12-tumor bearing mice. The uptake of ^211^At-MABG in tumors was higher than that in other organs and tissues at all time points (Table 1). ^211^At-MABG rapidly accumulated in tumors, and tumor uptake at 1 h after injection reached approximately 30% ID/g. The highest tumor uptake was reached at 3 h, and thereafter uptake decreased gradually. However, accumulation remained high at 24 h. PC12 tumors showed high absorbed doses of ^211^At-MABG (10.21 Gy/MBq; Table 2). Compared with other normal organs and tissues, the adrenal gland and heart with high norepinephrine transporter expression showed relatively high uptake (Table 1). The estimated doses absorbed by the adrenal gland and heart were 5.07 and 4.08 Gy/MBq, respectively (Table 2).

**Table 1:** Biodistribution of ^211^At-MABG in PC12 tumor-bearing mice

Organ	^211^At-MABG uptake				
	1 h	3 h	6 h	12 h	24 h
Blood	0.83 ± 0.05	0.53 ± 0.04	0.41 ± 0.04	0.39 ± 0.07	0.25 ± 0.07
Liver	9.79 ± 1.44	5.30 ± 0.57	3.17 ± 0.30	2.22 ± 0.34	1.05 ± 0.32
Kidney	2.88 ± 0.23	1.83 ± 0.06	1.65 ± 0.19	1.24 ± 0.30	0.77 ± 0.15
Adrenals	14.51 ± 4.32	10.71 ± 2.96	20.77 ± 8.22	9.11 ± 2.60	10.43 ± 1.59
Intestine	7.18 ± 0.73	4.32 ± 0.22	3.99 ± 0.69	2.93 ± 0.91	1.74 ± 0.50
Spleen	6.82 ± 1.23	6.27 ± 0.85	6.42 ± 0.82	4.43 ± 0.93	3.08 ± 0.87
Pancreas	7.62 ± 0.86	3.89 ± 0.84	2.22 ± 0.49	1.68 ± 0.45	0.95 ± 0.21
Stomach	3.41 ± 1.04	2.41 ± 0.44	5.08 ± 1.08	6.71 ± 0.76	3.39 ± 1.10
Heart	18.49 ± 1.54	11.61 ± 2.99	7.35 ± 1.89	4.94 ± 1.56	2.66 ± 0.32
Lung	8.49 ± 2.00	4.57 ± 1.21	2.68 ± 1.22	2.67 ± 0.63	1.64 ± 0.77
Muscle	2.15 ± 0.14	1.59 ± 0.28	1.20 ± 0.17	0.88 ± 0.21	0.42 ± 0.17
Bone	2.24 ± 0.42	1.20 ± 0.20	0.86 ± 0.27	0.74 ± 0.10	0.38 ± 0.27
Brain	0.13 ± 0.04	0.08 ± 0.01	0.04 ± 0.01	0.07 ± 0.03	0.03 ± 0.01
PC12 tumor	29.10 ± 9.31	36.21 ± 16.74	22.85 ± 10.69	28.39 ± 14.94	16.43 ± 5.69
Thyroid	1.52 ± 0.94	1.65 ± 0.97	0.72 ± 0.47	0.61 ± 0.40	0.61 ± 0.19

The values presented are mean ± SD percentage injected radioactivity dose per gram (% ID/g), except the thyroid values which are percentage injected radioactivity dose (% ID)

**Table 2:** Estimated absorbed doses of ^211^At-MABG

Organ	Absorbed dose (Gy/MBq)
Blood	0.18
Liver	1.41
Kidney	0.61
Adrenals	5.07
Intestine	1.45
Spleen	2.32
Pancreas	1.28
Stomach	1.46
Heart	4.08
Lung	1.63
Thyroid	1.49
Muscle	0.52
Bone	0.47
Brain	0.02
PC12 tumor	10.21

#### Tumor volume reduction after ^211^At-MABG treatment

**Fig. 2:**
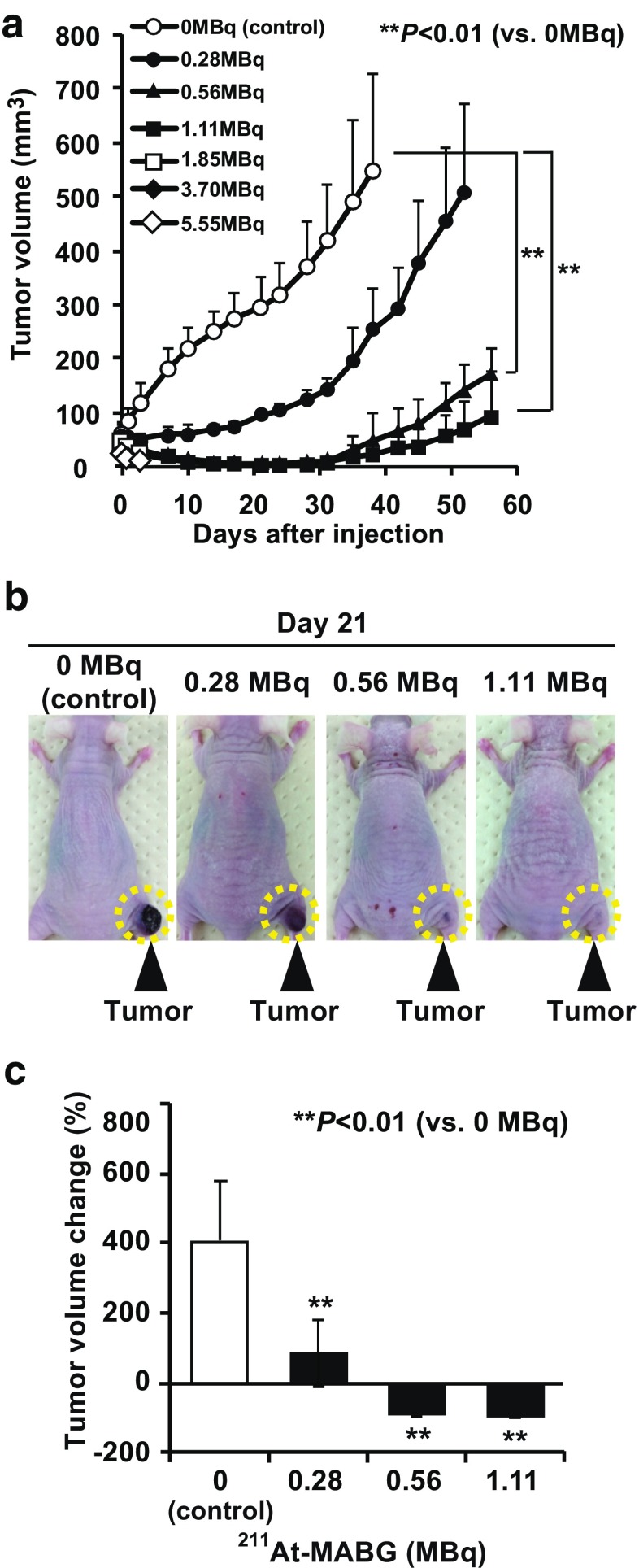
Effects of ^211^At-MABG on tumor volume in PC12 tumor-bearing mice. **a** Tumor growth curves after treatment with ^211^At-MABG (five mice per group). **p < 0.01 vs. 0 MBq (control). **b** Representative images of mice on day 21 after treatment with ^211^At-MABG and control (0 MBq). *Dashed circles* indicate tumors. **c** Tumor volume percentage change on day 21 after treatment with 0, 0.28, 0.56 and 1.11 MBq of ^211^At-MABG (five mice per group). **p < 0.01 vs. 0 MBq

There were no significant differences in tumor volumes at baseline (day 0) among the control group and the six ^211^At-MABG treatment groups. The control group showed rapid growth of PC12 tumors (48.9 ± 7.7 mm^3^ on day 0 to 591.8 ± 178.1 mm^3^ on day 21, *p* < 0.01). The ^211^At-MABG treatment groups showed significant dose-dependent reductions in tumor volume (Fig. 2a). In mice injected with 0.28 MBq of ^211^At-MABG tumor growth was suppressed until day 7 (58.2 ± 19.4 mm^3^ on day 0, 59.2 ± 16.8 mm^3^ on day 7, *p* = 0.844), but the tumors grew thereafter. In mice injected with 0.56 and 1.11 MBq of ^211^At-MABG, tumor volume was rapidly reduced. Thus, we observed reductions in tumor volumes on day 1 (Fig. 2a). The smallest tumor volumes were reached around day 21 (0.56 MBq, 48.9 ± 7.8 mm^3^ on day 0 to 4.5 ± 2.3 mm^3^ on day 21, *p* < 0.01; 1.11 MBq, 48.7 ± 8.0 mm^3^ on day 0 to 1.7 ± 1.9 mm^3^ on day 21, *p* < 0.01; Fig. 2a, b). Therefore, in the control group tumor volume increased by 409.% ± 169.1% from day 0 to day 21, whereas tumor volumes in the mice injected with 0.56 and 1.11 MBq of ^211^At-MABG decreased by 90.4 ± 5.5% and 96.7% ± 3.4% from day 0 to day 21, respectively (*p* < 0.01, vs. control; Fig. 2c). In two of the five mice injected with 1.11 MBq ^211^At-MABG, the tumor disappeared until around day 28.

#### Survival after ^211^At-MABG treatment

**Fig. 3:**
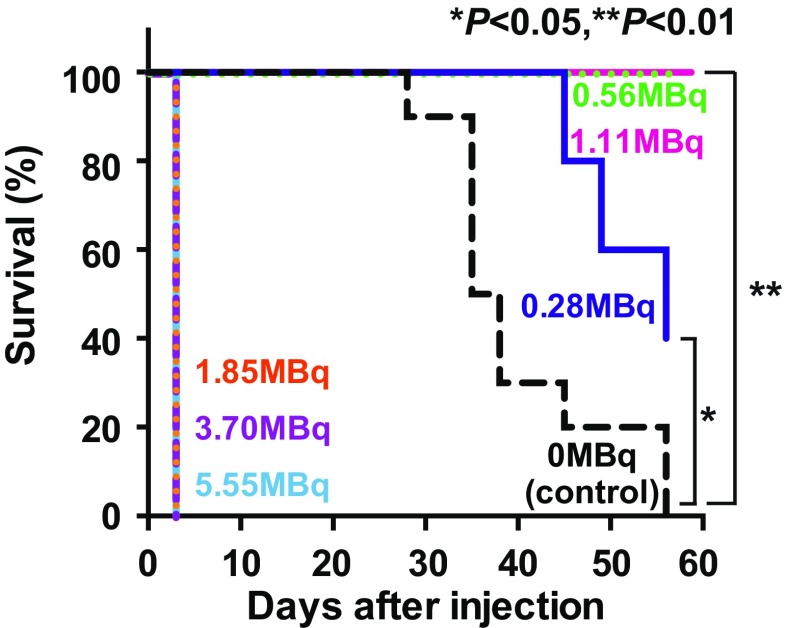
Kaplan-Meier survival curves. Endpoints were designated as an increase in tumor volume to 500 mm^3^ and a decrease in body weight by more than 20% from day 0. Groups treated with 0.56 and 1.11 MBq ^211^At-MABG showed significantly better survival than the control group. **p < 0.01, *p < 0.05, vs. 0 MBq

Figure 3 shows Kaplan-Meier survival curves based on the endpoints of tumor volume and weight loss. The control group showed a lower survival rate than the ^211^At-MABG administration groups (*p* < 0.05 for 0.28 MBq, *p* < 0.01 for 0.56 and 1.11 MBq).

### Absorbed radiation dose

The estimated radiation dose absorbed by PC12 tumors was 10.21 Gy/MBq (Table 2), and the calculated dose absorbed by tumors treated with 1.11 MBq of ^211^At-MABG was therefore 11.3 Gy. The efficacy of 1.11 MBq of ^211^At-MABG was almost equivalent to that of 30 Gy of external X-ray irradiation (Supplementary Fig. 4).

### Weight change after ^211^At-MABG treatment

**Fig. 4:**
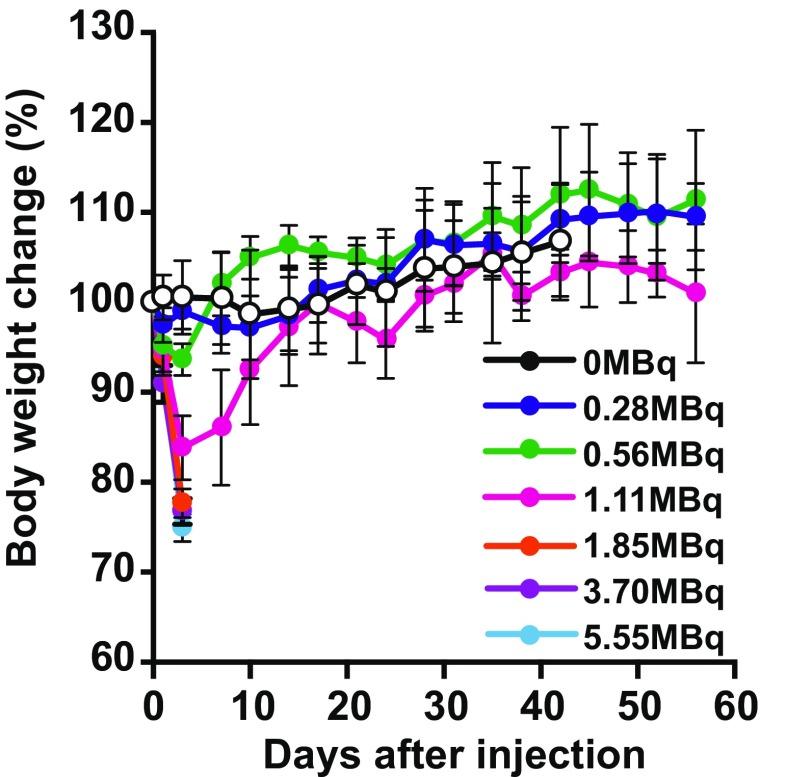
Body weight changes after treatment with ^211^At-MABG in PC12 tumor-bearing mice shown as means (*symbols*) with standard deviations (*error bars*; five mice per group)

^211^At-MABG treatment caused a dose-dependent decrease in body weight soon after administration (Fig. 4). On day 3 after injection, all the mice injected with 0.28 and 0.56 MBq of ^211^At-MABG showed a decrease in body weight of less than 5% (*p* ≤ 0.05 for 0.28 MBq, *p* ≤ 0.01 for 0.56 MBq). Also, mice injected with 1.11 MBq of ^211^At-MABG showed a decrease in body weight of 10–20% (*p* ≤ 0.01), and a decrease in body weight of 20% was observed in one of the five mice 3 days after injection. However, the body weight decrease in all these mice was temporary, and in these groups body weight gradually recovered. As a result, there were no differences in body weight of the mice treated with 0.28, 0.56 and 1.11 MBq ^211^At-MABG compared with that of the control group on day 10 after injection (*p* = 0.154).

In contrast, all mice treated with 1.85, 3.70 and 5.55 MBq ^211^At-MABG showed decreases in body weight of more than 20% on day 3 or 4 after ^211^At-MABG administration, and were therefore killed humanely at that time. Their tumors were resected and used for histological analysis. Based on these data, the maximum tolerated dose (MTD) of ^211^At-MABG in the nude mice was 1.11 MBq.

### Histological analysis of PC12 tumors after ^211^At-MABG treatment

**Fig. 5:**
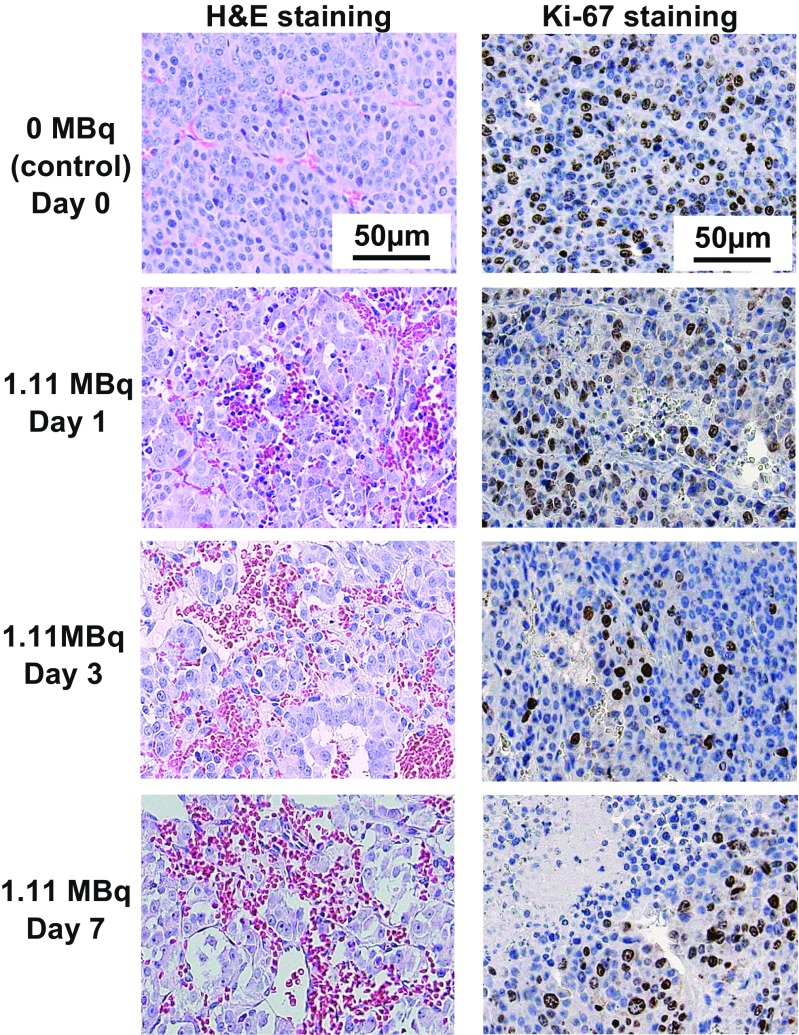
Temporal histological changes in PC12 tumors after administration of 1.11 MBq ^211^At-MABG. Representative images of H&E-stained sections (*left*) and Ki-67-stained sections (*right*) of PC12 tumors on days 1, 3 and 7 after administration of 1.11 MBq of ^211^At-MABG (*scale bars* 50 μm)

Since 1.11 MBq of ^211^At-MABG was the MTD, we analyzed the temporal histological changes in the PC12 tumors on days 1, 3 and 7 after administration of 1.11 MBq ^211^At-MABG. Tumor sections were stained with H&E and Ki-67 as a proliferation marker. In sections of control tumors (0 MBq), the cells were arranged in a nest pattern and were surrounded by fibrovascular stroma (Fig. 5, left upper panel). In sections of tumors from mice treated with ^211^At-MABG, there were no nests of tumor cells, whereas hemorrhage and lymphocyte infiltration were observed (Fig. 5, left panels). The hemorrhage increased in a time-dependent manner, and a small necrotic area was observed on day 3, and the area had expanded by day 7 (Fig. 5, left panels). In Ki-67-stained sections of tumors from mice treated with ^211^At-MABG, proliferating (Ki-67-positive) tumor cells tended to decrease in a time-dependent manner (Fig. 5, right panels).

**Fig. 6:**
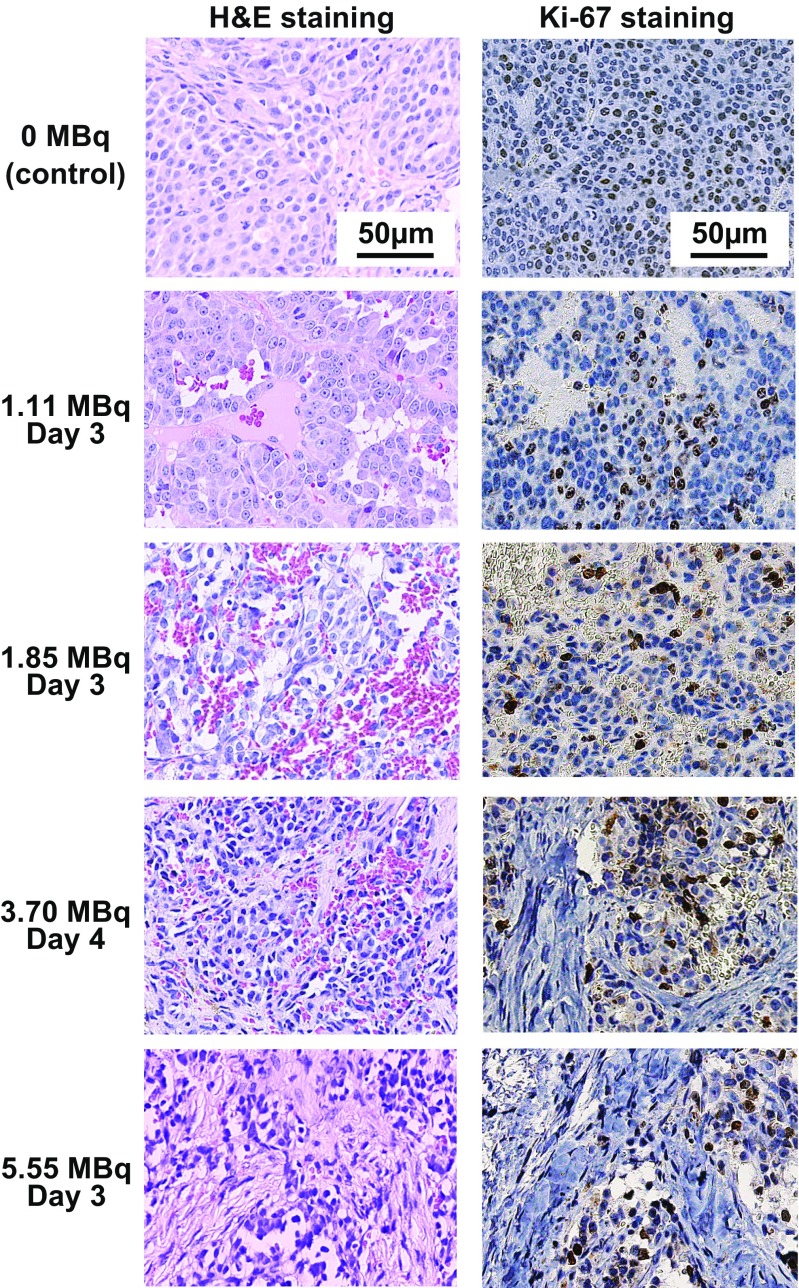
Dose-dependent histological changes in PC12 tumors after administration of ^211^At-MABG. Representative images of H&E-stained sections (*left*) and Ki-67-stained sections (*right*) of PC12 tumors on day 3 or 4 after administration of 1.11, 1.85, 3.70 and 5.55 MBq ^211^At-MABG (*scale bars* 50 μm)

Sections of tumors from mice treated with 1.85 MBq ^211^At-MABG showed larger hemorrhage and necrotic areas than following treatment with 1.11 MBq ^211^At-MABG (Fig. 6, left panels). In sections of tumors from mice treated with 3.70 MBq ^211^At-MABG, partial replacement by fibrous tissue was also observed in addition to hemorrhage and necrosis (Fig. 6, left panels). The area of fibrous tissue was larger following treatment with 5.55 MBq ^211^At-MABG (Fig. 6, left panels). In Ki-67-stained sections, proliferating tumor cells tended to decrease in a time-dependent manner (Fig. 6, left panels).

### Histological changes in bone marrow, adrenal gland, and heart after ^211^At-MABG treatment

**Fig. 7:**
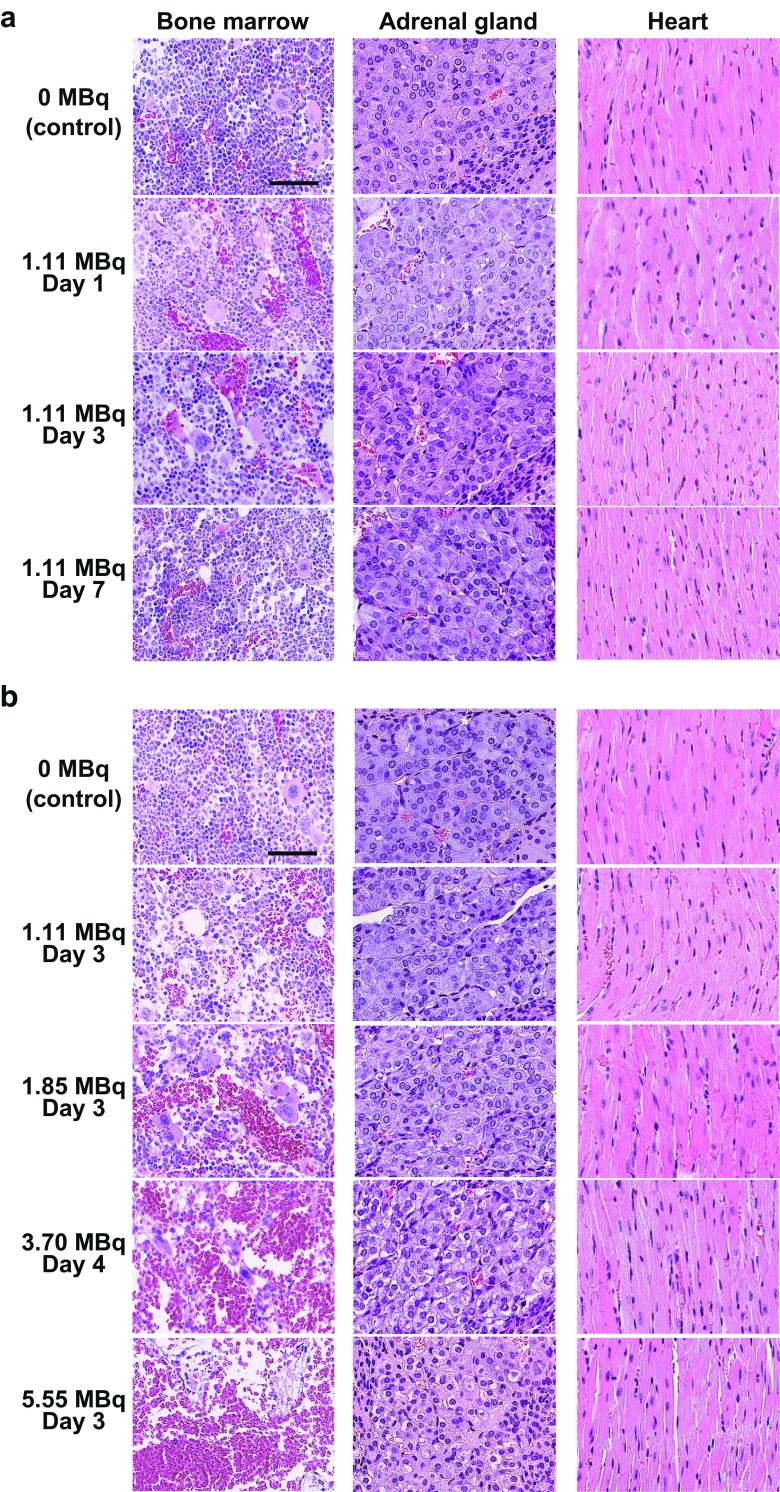
Histological changes in bone marrow, adrenal gland and heart after administration of ^211^At-MABG. Representative images of H&E-stained sections: **a** days 1, 3, and 7 after administration of 1.11 MBq ^211^At-MABG, **b** day 3 or 4 after administration of 1.11, 1.85, 3.70 and 5.55 MBq ^211^At-MABG (*scale bars* 50 μm)

In sections of bone marrow from mice treated with 1.11 MBq ^211^At-MABG, dilated vascular sinuses filled with erythrocytes were observed on day 1 after ^211^At-MABG administration. The number of myeloid cells showed a slight decrease on day 3. However, on day 7 after administration of 1.11 MBq ^211^At-MABG, the number of myeloid cells had recovered to the same level as in control mice (Fig. 7a). In the adrenal gland and the heart from mice treated with 1.11 MBq ^211^At-MABG, histological changes were not observed on days 1, 3 and 7 (Fig. 7a). Sections of femur from mice killed 3 or 4 days after administrations of 1.85, 3.70 and 5.55 MBq ^211^At-MABG showed decreases in bone marrow cellularity and increases in density of erythrocytes within the expanded vascular sinuses in a dose-dependent manner (Fig. 7b). In particular, administration of 5.55 MBq ^211^At-MABG induced severe depletion of cells in the bone marrow, vascular dilation and hemorrhage (Fig. 7b). Although histological changes were not observed in sections of adrenal glands from mice treated with 1.11 MBq and 1.85 MBq ^211^At-MABG, some vacuolated medullary cells were observed in the adrenal glands from mice treated with 3.70 and 5.55 MBq ^211^At-MABG (Fig. 7b). Sections of heart from mice treated with 1.85, 3.70 and 5.55 MBq ^211^At-MABG and mice in the control group showed no differences in morphological features (Fig. 7b).

## Discussion

Treatment with ^211^At-MABG reduced the tumor volumes in PC12 tumor-bearing mice in a dose-dependent manner. In mice treated with ^211^At-MABG, reductions in body weight and in the number of myeloid cells in the bone marrow were not severe. This may indicate that ^211^At-MABG has tumor-reducing effects without severe radiation-induced side effects.

Histology showed hemorrhage and tumor necrosis soon after ^211^At-MABG administration. These histological findings confirm the tumor volume-reducing effects of ^211^At-MABG in in vivo studies.

### Quality of ^211^At-MABG

The radiochemical purity of ^211^At-MABG was more than 99.7%. In addition, the selective norepinephrine transporter inhibitor DMI as well as norepinephrine inhibited cell uptake of the radiolabeled product. These results agree with those of previous studies involving neuroblastoma cells [29, 30], and indicate that the quality of the ^211^At-MABG used in this study was appropriate.

### In vitro tumor cell growth suppression effects of ^211^At-MABG

In this study, ^211^At-MABG reduced the PC12 cell survival ratio in a dose-dependent manner. This effect of ^211^At-MABG in reducing cell survival agrees with the findings of previous in vitro studies looking at the toxicity of ^211^At-MABG in neuroblastoma cells [20]. This finding further expands the possible role of ^211^At-MABG in the treatment of pheochromocytoma.^211^At-MABG induced LDH release as a cell-death marker and dose-dependently increased the number of cells with DNA DSB. These results suggest that decreased cell survival following ^211^At-MABG treatment is probably due to cell death induced by DNA DSB. This cell death mechanism, as confirmed in this study, supports the hypothesis of cell injury by α-particles and is most likely the main mechanism by which ^211^At-MABG causes cell death in pheochromocytoma [16, 31].

### Therapeutic effects of ^211^At-MABG in PC12 tumor-bearing mice

Similar to the findings of a previous study using a neuroblastoma model [20], biodistribution studies showed that ^211^At-MABG accumulated more in the adrenal gland and heart than in other organs. Both the adrenal gland and heart have a rich sympathetic nervous system. Therefore, a norepinephrine analog may accumulate more readily in these organs [32, 33]. The current data also show very high ^211^At-MABG uptake (about 35% ID/g) in PC12 tumors. A biodistribution study of ^211^At-MABG in a neuroblastoma mouse model (SK-N-SH xenograft) showed about 4% ID/g uptake by SK-N-SH tumors [32]. Although we did not directly compare the therapeutic effects of ^211^At-MABG in the neuroblastoma model to those in pheochromocytoma models (because we have not yet been able to establish a neuroblastoma mouse model), the current data suggest that ^211^At-MABG may be more effective in pheochromocytoma than in neuroblastoma. This possibility should be investigated in future studies.

Some previous studies investigating neuroblastoma and pheochromocytoma cells in vitro have shown the possible therapeutic effects of ^211^At-MABG [20]. However, there have been no studies looking at the antitumor therapeutic effects of ^211^At-MABG in neuroblastoma and pheochromocytoma animal models in vivo. In this regard, this study showed that ^211^At-MABG has therapeutic effects in vivo, and thus provides further insight into the therapeutic potential of ^211^At-MABG.

In PC12 tumor-bearing mice, ^211^At-MABG treatment at doses of 0.56 MBq and higher reduced tumor volumes compared with those in control mice. The present study once again provides new insights into the therapeutic effects of ^211^At-MABG in a pheochromocytoma mouse model. ^211^At-MABG administration led to almost complete disappearance of tumor for up to 21 days. The protocol used in this study involved a single administration of ^211^At-MABG. Thus, after 21 days, tumor cells began to grow. In the clinical setting, ^131^I-MIBG is usually administered at intervals of 6 weeks to 3 months [10, 11]. The lack of repeated ^211^At-MABG administrations is one of the limitations of this study; future studies should focus on the effects of repeated treatments.

### Histological findings on the therapeutic effects of ^211^At-MABG

The histological findings of this study confirmed the effectiveness of ^211^At-MABG in reducing tumor volumes. Clinical research using ^131^I-MIBG has shown that most patients achieve stable disease, and that complete remission was very rare [4, 13]. These clinical data suggest that the cytotoxic effects of the β-emitting ^131^I-MIBG are limited. In contrast, in this study the PC12 tumors in mice receiving higher doses of ^211^At-MABG showed necrosis and fibrosis at earlier time points than tumors in mice receiving lower doses. Although careful dose setting would be necessary, it is possible that ^211^At-MABG treatment could lead to complete remission in patients with malignant pheochromocytoma. In contrast, catecholamine released from damaged pheochromocytoma cells can result in catecholamine crisis. This causes hypertension and catecholamine-induced cardiomyopathy [5, 34]. Patient management after ^211^At-MABG therapy should be considered as the next step prior to clinical trials.

### Possible therapeutic effects of ^211^At-MABG versus ^131^I-MIBG

In this study, we could not directly compare the therapeutic effects of ^211^At-MABG with those of ^131^I-MIBG. However, in a study by Rutgers et al. using the same pheochromocytoma mouse model, administration of 57 MBq of ^131^I-MIBG maximally reduced tumor volume to approximately 30% of the volume on day 0 [35], while in this study tumors treated with 1.11 MBq ^211^At-MABG were reduced to approximately 3.3% of the volume on day 0. Therefore, the MTD of ^211^At-MABG, while being a fraction of the MTD for ^131^I-MIBG, would have a significantly greater tumor-reducing effect (approximately nine times) in pheochromocytoma therapy than ^131^I-MIBG.

A systematic review and meta-analysis of the effect of ^131^I-MIBG on tumor volume found a complete remission rate after ^131^I-MIBG therapy of 3%, a partial remission rate of 27% and a stable disease rate of 52% [13]. In this study, all five mice treated with the MTD of ^211^At-MABG (1.11 MBq) showed a reduction in tumor volume, and in two of them the tumor disappeared until day 28 after ^211^At-MABG administration. Moreover, based on the percentage tumor volume reduction, in mice receiving 1.11 MBq ^211^At-MABG, tumor volumes were reduced by 96.7% compared to the volumes at baseline. Strictly speaking, although the RECIST criteria cannot be applied to our data [36], in mice treated with the MTD of ^211^At-MABG (1.11 MBq), 40% achieved complete remission and 60% had partial remission. In terms of side effects of ^131^I-MIBG, the most frequently reported side effects were hematologic toxicity with grade 3 or 4 neutropenia that occurred in up to 87% and grade 3 or 4 thrombocytopenia that occurred in up to 83% [13]. Although we did not monitor complete blood cell counts before and after ^211^At-MABG administration, bone marrow histological findings revealed rapid recovery of bone marrow cellularity. Therefore, ^211^At-MABG may have greater therapeutic effects with fewer side effects than ^131^I-MIBG.

### Safety aspects of ^211^At-MABG therapy

One of the greatest limitations of current TRT is radiation-related side effects such as bone marrow suppression. Since β-emitting radiotracers have far-reaching effects, surrounding organs are irradiated and damaged [9]. On the other hand, because of the short range of α-particles (<100 μm) radiation-induced side effects are minimized in the clinical setting [37]. In this study, we measured the body weight of the mice to monitor radiation-induced adverse effects and, based on the body weight reduction, decided that the MTD of ^211^At-MABG in nude mice was 1.11 MBq. Mice receiving a dose of ^211^At-MABG lower than or equal to the MTD showed temporary weight reduction after administration but then a gradual recovery in body weight. Thus, there was no significant weight reduction at 10 days after ^211^At-MABG administration compared with the body weight of control mice.

Changes in bone marrow cellularity indicate systemic toxicity from exposure to chemicals or radiation. Therefore, evaluation of bone marrow is important in toxicity and safety assessments [38, 39]. We evaluated bone marrow cellularity using H&E-stained femur sections. The treatment with ^211^At-MABG at the MTD (1.11 MBq) caused no marked change in bone marrow cellularity compared with the bone marrow of the control group. In contrast, treatment with 1.85, 3.70 and 5.55 MBq of ^211^At-MABG induced obvious decreases in the number of nucleated cells and increases in the density of erythrocytes within the dilated vascular spaces. Sections of the adrenal gland and heart that express norepinephrine transporter were also evaluated. There were no marked histological changes in any organs treated with the MTD of ^211^At-MABG, whereas some vacuolated medullary cells were detected in adrenal gland treated with 3.70 and 5.55 MBq of ^211^At-MABG. These results indicate that the MTD of ^211^At-MABG determined in terms of body weight should be safe based on these histological findings.

### Study limitations

The protocol used in this study involved a single ^211^At-MABG administration because the aim was to clarify the initial therapeutic effects and to evaluate the adverse effects. Based on the current data, we determined the therapeutic MTD of ^211^At-MABG. The next step will be to conduct a repeated-treatment study in the near future. We evaluated the therapeutic and adverse effects of ^211^At-MABG but did not directly compare these with the effects of ^131^I-MIBG. We compared the therapeutic efficacy of ^211^At-MABG with that of ^131^I-MIBG in previously published studies, and we estimated the effective dose of ^211^At-MABG.

## Conclusions

^211^At-MABG showed a strong tumor volume-reducing effect in a pheochromocytoma mouse model without severe adverse effects such as weight reduction and reductions in the numbers of myeloid cells in the bone marrow. Therefore, ^211^At-MABG might be an effective therapeutic agent for the treatment of malignant pheochromocytoma.

## Electronic supplementary material

ESM 1 (DOC 55 kb)

Supplementary Fig. 1 Retention times of ^211^At-MABG and nonradioactive MIBG (19.1 min and 18.7 min, respectively) (JPEG 103 kb)

High Resolution (EPS 526 kb)

Supplementary Fig. 2 Uptake of ^211^At-MABG by PC12 cells with high norepinephrine transporter expression. **a** The cell uptake assay shows that ^211^At-MABG was rapidly transported into the cells. **a** The inhibition assay shows that desipramine (DMI), a selective inhibitor of the norepinephrine transporter, and *dl*-norepinephrine (NE) significantly inhibited cell uptake of ^211^At-MABG (*p* < 0.01) (JPEG 115 kb)

High Resolution (EPS 627 kb)

Supplementary Fig. 3 Induction of lactate dehydrogenase (LDH) release (a cell death marker) from PC12 cells following ^211^At-MABG administration (**p < 0.01) (JPEG 132 kb)

High Resolution (EPS 479 kb)

Supplementary Fig. 4 Effect of various doses of ^211^At-MABG on tumor volume. The calculated dose absorbed by tumors treated with 1.11 MBq of ^211^At-MABG was 11.3 Gy, and the effect of 1.11 MBq was almost equivalent to that of 30 Gy of external X-ray irradiation (JPEG 82 kb)

High Resolution (EPS 576 kb)
