# Supplementary material for: Antitumor effects of radionuclide treatment using α-emitting meta-211At-astato-benzylguanidine in a PC12 pheochromocytoma model
Source: Eur J Nucl Med Mol Imaging. 2018 Jan 19;45(6):999–1010. doi: 10.1007/s00259-017-3919-6 (PMC5915519; doi:10.1007/s00259-017-3919-6)
Supplement: Supplementary file 1 — (DOC 55 kb) [file 259_2017_3919_MOESM1_ESM.doc]

**Supplementary information for "Antitumor effects of radionuclide treatment using α-emitting *meta*-^211^At-astato-benzylguanidine in a PC12 pheochromocytoma model"**

**Supplemental materials and methods**

Production of ^211^At and Radiosynthesis of ^211^At-MABG

^211^At was produced via the ^209^Bi(α,2n)^211^At reaction . We obtained ^209^Bi foil (size: 10 mm × 10 mm × 0.25 mm thickness, purity: 99.999%) from Goodfellow Corporation (Coraopolis, PA, USA). ^209^Bi foil was irradiated with alpha particles (50 MeV) for up to 3 h using an azimuthally varying-field cyclotron at our institutions. The on-target particle energy was 28.1 MeV degraded by 0.4 mm thickness of Al plate. Typical beam current used in this study was 3.5 µA. ^211^At in the activated target was recovered by the dry distillation method followed by previous reports with some modifications. Briefly, an irradiated target was heated on a quartz boat at 650°C for 30 min under streaming of He gas (40 cc/min) to distill ^211^At, and the stream was introduced into a polyetheretherketone (PEEK) tube to trap volatile ^211^At. After cooling to room temperature, ^211^At trapped inside the PEEK tube was harvested with chloroform (500 µl, flow rate: 250 µl/min.). Radioactivity and radionuclidic purity were evaluated by γ-ray spectrometry using an HP-Ge detector coupled to a multichannel analyzer (Seiko EG&G Orsim MCA 7700) and a gamma energy of 682 keV for identification of ^211^At. Harvested radioactivity of ^211^At was 22.1–93.2 MBq at the end of bombardment. ^211^At-MABG was synthesized using modifications of previously reported procedures . The chloroform solution containing ^211^At was added in a reaction vial and then removed by evaporation with a gentle stream of N_2_ at room temperature. To the vial were added *meta*-trimethylsilylbenzylguanidine hemisulfate/methanol solution (0.2 mg, 0.74 µmol) and *N*-chlorosuccinimide/methanol solution (0.4 µg, 3 µmol). After removing methanol with a gentle stream of N_2_, 100 µl of trifluoroacetic acid (TFA) was added to the residue. The mixture was then heated at 70°C for 10 min. After cooling to room temperature, TFA was evaporated by a gentle stream of N_2_ gas. Residue in the vial was dissolved in 150 µl of methanol and then diluted with 400 µl of H_2_O. The diluted solution was injected into reversed-phase (RP) radio-high-performance liquid chromatography (radio-HPLC). A radioactive peak having a retention time close to that of the corresponding non-radioactive *meta*-iodobenzylguanidine (MIBG) was fractionated. After fractions were concentrated, the mixture was diluted with phosphate buffered saline (PBS) for subsequent studies. Harvested radioactivity was measured by a high-purity germanium (HP-Ge) detector, and radiochemical purity was determined by RP radio-HPLC.

Cell uptake and inhibition assay

For cell uptake, 1 × 10^5^ cells were incubated with 5.0 kBq of ^211^At-MABG in growth medium for 0.5, 1, 3, 6, 12 and 24 hours. After being washed with ice-cold PBS, cells were dissolved in 0.1 N NaOH. The radioactivity of ^211^At-MABG was measured by γ-counter. For inhibition assay, 1 × 10^5^ cells were incubated with 5.0 kBq of ^211^At-MABG in growth medium with or without 100 mM of desipramine (DMI) or 200 mM of *dl*-norepinephrine (NE) (Sigma-Aldrich, St. Louis, MO, USA). After incubation at 37°C or 4°C for 10 min, cells were washed with ice-cold PBS and dissolved in 0.1 N NaOH. The radioactivity of ^211^At-MABG was measured by γ-counter. The uptake of ^211^At-MABG was normalized radioactivity of control cells.

Lactate dehydrogenase (LDH) release assay

Cells (1 × 10^4^ cells/well) were incubated for 24 h in a 96-well culture plate and treated with 0.6 kBq/mL of ^211^At-MABG for 24 or 48 h. At the end of incubation, supernatants were collected and the LDH content was measured using a Cytotoxicity Detection Kit (Roche Diagnostics, Laval, Quebec, Canada). LDH release is expressed as percentage of total content, which was determined by lysing an equal number of cells with 1% Triton X-100.

Dosimetry

The mean absorbed dose per unit of injected ^211^At-MABG activity was calculated according to the following formula: D = A ̃∙E∙φ/m, where A ̃, the cumulated activity or time-integrated activity from zero to infinity in the organ or tumor, is equal to Te∙A0/ln (2); E is the mean emitted energy per α-disintegration, 6.9 MeV/Bq/s, calculated from the complex disintegration of ^211^At and ^211^Po ; φ is the absorbed fraction set to 1.0, and m is the mean weight of the organ or tumor collected in the biodistribution experiment. Te is the term for the effective half-life based on physical decay, biological clearance and uptake. A0 is the activity in the organ or tumor at time zero. Te and A0 were estimated from the biodistribution data using the nonlinear exponential curve fitting with the Levenberg-Marquardt algorithm (Origin, MicroCal Software, Inc., MA, USA).

External X-ray irradiation in a PC12 pheochromocytoma model

When tumor volume reached approximately 50 mm^3^, PC12 tumors (*n* = 5 each dose) were irradiated with 0, 5, 15, 30 and 60 Gy of X-rays at a rate of 4.4 Gy/min with a TITAN-320 X-ray generator (Shimadzu, Kyoto, Japan). The other parts of the mouse body were covered with a brass shield to limit unnecessary radiation exposure. The tumor size and body weight were measured at least twice a week for 8 weeks. The tumor size was measured using a digital caliper, and the tumor volume was calculated using the following formula: tumor volume (mm^3^) = (length × width^2^)/2. When body weight loss showed more than 20% compared with that at day 0, moribund state signs were observed, or the tumor volume reached 800 mm^3^, the mouse was euthanized humanely by isoflurane inhalation.

**Supplemental references**

1. Nagatsu K, Minegishi K, Fukada M, Suzuki H, Hasegawa S, Zhang MR. Production of (211)At by a vertical beam irradiation method. Appl Radiat Isot. 2014;94:363-71. doi:10.1016/j.apradiso.2014.09.012.

2. Vaidyanathan G, Zalutsky MR. 1-(m-[211At]astatobenzyl)guanidine: synthesis via astato demetalation and preliminary in vitro and in vivo evaluation. Bioconjug Chem. 1992;3:499-503.

3. Spetz J, Rudqvist N, Forssell-Aronsson E. Biodistribution and dosimetry of free 211At, 125I- and 131I- in rats. Cancer Biother Radiopharm. 2013;28:657-64. doi:10.1089/cbr.2013.1483.

**Supplemental figure legends**

**Supplemental Fig.1.** **Chromatogram of ^211^At-MABG (left) and MIBG (right) analyzed**

**by HPLC.**

**Supplemental Fig. 2. Cell uptake and inhibition assays in PC12 pheochromocytoma cells. (a)** Temporal cell uptake of ^211^At-MABG in PC12 cells. **(b)** Inhibition of ^211^At-MABG uptake in PC12 cells. The uptake of ^211^At-MABG was inhibited by desipramine (DMI), *dl*-norepinephrine (NE) and incubation at 4°C. Symbols indicate mean, and error bars indicate SD.

**Supplemental Fig. 3. Cytotoxicity of ^211^At-MABG in PC12 pheochromocytoma cells.** Lactate dehydrogenase (LDH) release as a cell-death marker was determined at 24 and 48 h after ^211^At-MABG treatment. Symbols indicate mean, and error bars indicate SD. ***p* < 0.01, vs. 0 kBq/mL.

**Supplemental Fig. 4.** **Therapeutic effects of external X-rays.** BALB/c-nu/nu mice bearing PC12 tumors were irradiated with X-rays. Symbols indicate mean, and error bars indicate SD. ***p* < 0.01, vs. 0 Gy.
