# Supplementary figures and images for: Antitumor effects of radionuclide treatment using α-emitting meta-211At-astato-benzylguanidine in a PC12 pheochromocytoma model
Source: Eur J Nucl Med Mol Imaging. 2018 Jan 19;45(6):999–1010. doi: 10.1007/s00259-017-3919-6 (PMC5915519; doi:10.1007/s00259-017-3919-6)

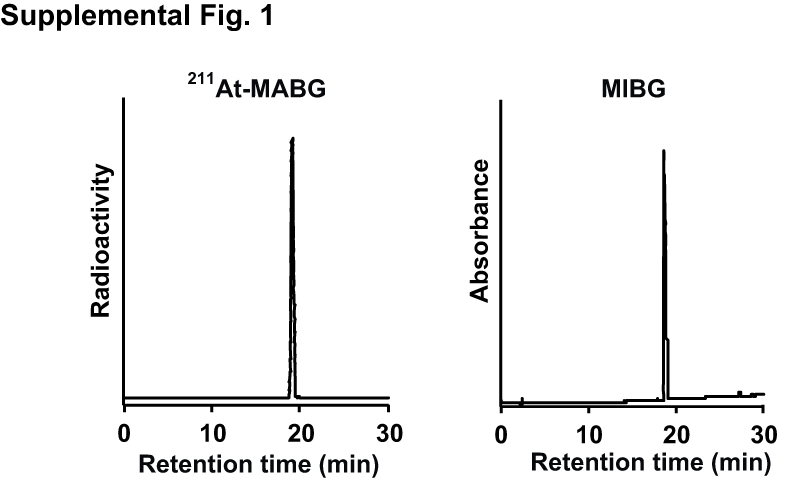

Supplement: Supplementary file 2 — Retention times of 211At-MABG and nonradioactive MIBG (19.1 min and 18.7 min, respectively) (JPEG 103 kb) [file 259_2017_3919_Fig8_ESM.jpg]

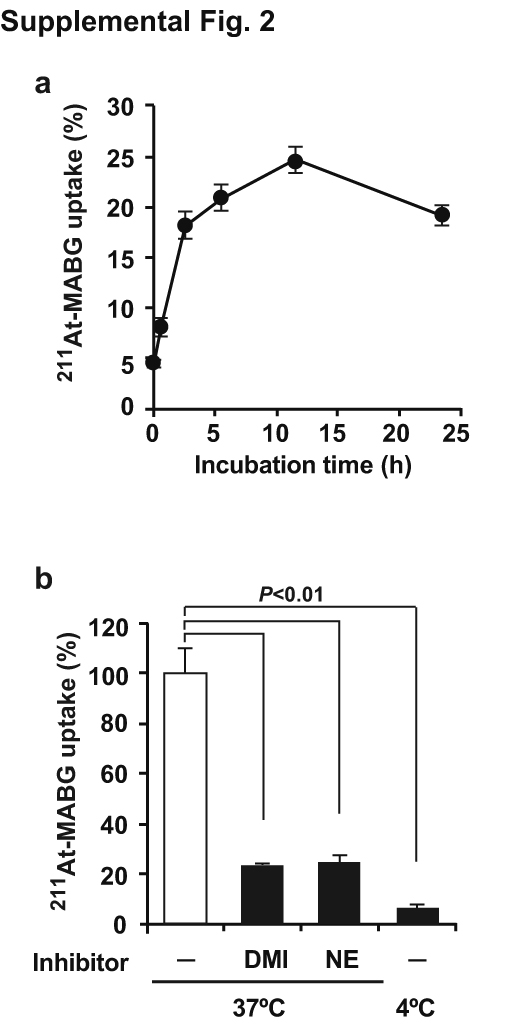

Supplement: Supplementary file 4 — Uptake of 211At-MABG by PC12 cells with high norepinephrine transporter expression. a The cell uptake assay shows that 211At-MABG was rapidly transported into the cells. a The inhibition assay shows that desipramine (DMI), a selective inhibitor of the norepinephrine transporter, and dl-norepinephrine (NE) significantly inhibited cell uptake of 211At-MABG (p < 0.01) (JPEG 115 kb) [file 259_2017_3919_Fig9_ESM.jpg]

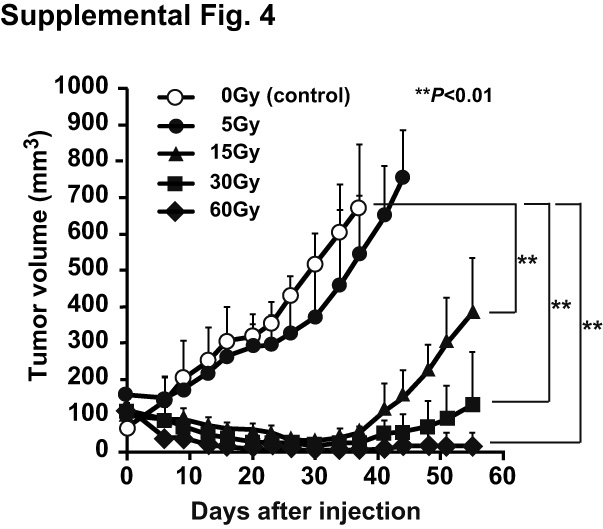

Supplement: Supplementary file 6 — Induction of lactate dehydrogenase (LDH) release (a cell death marker) from PC12 cells following 211At-MABG administration (**p < 0.01) (JPEG 132 kb) [file 259_2017_3919_Fig11_ESM.jpg]

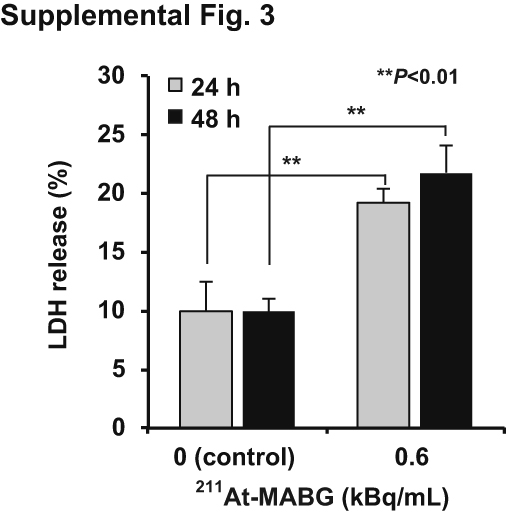

Supplement: Supplementary file 8 — Effect of various doses of 211At-MABG on tumor volume. The calculated dose absorbed by tumors treated with 1.11 MBq of 211At-MABG was 11.3 Gy, and the effect of 1.11 MBq was almost equivalent to that of 30 Gy of external X-ray irradiation (JPEG 82 kb) [file 259_2017_3919_Fig10_ESM.jpg]
